# The Sexual Dimorphism of the Neuroimmune Response in the Brains of *Taenia crassiceps*-Infected Mice

**DOI:** 10.3390/brainsci14111127

**Published:** 2024-11-08

**Authors:** Karen Elizabeth Nava-Castro, Diana Lizeth Ruiz-Antonio, María del Sol Ríos-Avila, Claudia Angélica Garay-Canales, Lenin Pavón, Romel Hernandez-Bello, Víctor Hugo Del Río-Araiza, Manuel Iván Girón-Pérez, Jorge Morales-Montor

**Affiliations:** 1 Grupo de Biología y Química Atmosféricas, Departamento de Ciencias Ambientales, Instituto de Ciencias de la Atmósfera y Cambio Climático, Universidad Nacional Autónoma de México, Ciudad de México 04510, Coyoacán, Mexico; karlenc@atmosfera.unam.mx (K.E.N.-C.); maria_rios@ciencias.unam.mx (M.d.S.R.-A.); 2 Departamento de Inmunología, Instituto de Investigaciones Biomédicas, Universidad Nacional Autónoma de México, Ciudad de México 04510, Coyoacán, Mexico; dializ1998@gmail.com (D.L.R.-A.); clausgaray@iibiomedicas.unam.mx (C.A.G.-C.); 3 Posgrado en Ciencias Bioquímicas, Universidad Nacional Autónoma de México, Unidad de Posgrado, edif D, 1er piso, Circuito de Posgrados, Ciudad Universitaria, Ciudad de México 04510, Coyoacán, Mexico; 4 Posgrado en Ciencias Biológicas, Universidad Nacional Autónoma de México, Unidad de Posgrado, edif D, 1er piso, Circuito de Posgrados, Ciudad Universitaria, Ciudad de México 04510, Coyoacán, Mexico; 5 Laboratorio de Psicoinmunología de la Dirección de Investigaciones en Neurociencias, Instituto Nacional de Psiquiatría “Ramón de la Fuente Muñiz”, Ciudad de México 14370, Tlalpan, Mexico; lkuriaki@inprf.gob.mx; 6 Departamento de Microbiología, Facultad de Medicina, Universidad Autónoma de Nuevo León, Monterrey 64260, Nuevo León, Mexico; romel.hernandezbl@uanl.edu.mx; 7 Departamento de Parasitología, Facultad de Medicina Veterinaria y Zootecnia, Universidad Nacional Autónoma de México, Ciudad de México 04510, Coyoacán, Mexico; victordelrioa@fmvz.unam.mx; 8 Laboratorio de Inmunotoxicología, Secretaría de Investigación y Posgrado, Universidad Autónoma de Nayarit, Tepic 63184, Nayarit, Mexico; ivangiron@uan.edu.mx

**Keywords:** sexual dimorphism, neuroinflammation, neurotransmitters, cytokines, cysticercosis, *Taenia crassiceps*, neuroimmunoendocrine network

## Abstract

Background: Helminth infections are associated with cognitive deficits, especially in school-age children. Deworming treatment in heavily infected children improves their short- and long-term memory recall. In mice, intraperitoneal helminth infection with *Taenia crassiceps* (*T. crassiceps*) shows sexual dimorphism in terms of the parasite load, immune response, hormone levels, and behavioral changes. We have previously shown poorer short-term memory performance and changes in the concentrations of cytokines and neurotransmitters in the hippocampus, which were replicated in this study. The molecular changes in other brain structures, such as those related to reproduction, are unknown. Methods: Male and female Balb/cAnN mice were chronically infected with *T. crassiceps* larvae. We determined the peritoneal parasite load and established the presence of cytokines and neurotransmitters in the hippocampus, olfactory bulb, and hypothalamus. Results: The parasite load was higher in female than male infected mice, as expected. In the hippocampus, the neurotransmitters norepinephrine and serotonin increased in males but decreased in females. In contrast, in the olfactory bulb and hypothalamus, the neurotransmitters assessed showed no statistical differences. The cytokine profiles were different in each brain structure. The TNF-α levels in the olfactory bulb and the IL-4 levels in the hippocampus of infected mice were dimorphic; IFN-γ was augmented in both male and female infected animals, although the increase was higher in infected males. Conclusions: The brain responds to peripheral infection with cytokine levels that vary from structure to structure. This could be a partial explanation for the dimorphic behavioral alterations associated with infection, it also demonstrates the synergic interaction between the immune, the endocrine, and the nervous systems.

## 1. Introduction

Cognitive deficits are associated with parasitic infections, with school-age children being the most vulnerable population [1]. This is especially true for those living in rural areas [2,3]. Although there are no studies providing direct evidence of this, eliminating the parasite, *Trichuris trichiura*, in heavily infected children improved their performances in terms of the auditory short-term memory and the retrieval of long-term memory [4]. Furthermore, a meta-analysis including the data of more than 12,000 children from 36 studies showed that soil-transmitted helminth-infected children without anthelmintic treatment present small or moderate deficits in terms of learning, memory, reaction time, and intelligence [5]. Sterile inflammation, presenting as the measurement of increased IL-6 levels in the serum of healthy 9-year-old children, was correlated with a poor performance in working memory [6].

The active immune response against infection triggers multiple mechanisms that modulate neuroendocrine signaling, which mainly consists of the interaction between cytokines, hormones, and neurotransmitters. These interactions are recognized as comprising the neuroimmune endocrine (NIE) network [6]. The NIE is affected by parasites, as shown in multiple studies. For example, many helminths and protozoa agents alter sexual hormonal levels, as reviewed by Gomez de León, who observed that *Toxoplasma gondii* infection increased testosterone levels. This is shown in several studies in humans and animal models [7]. In contrast, plasmodium infection reduces the testosterone levels [8]. Regarding neurotransmitter modulation, in a mice model of *Trypanosoma cruzi* infection, there is a significant reduction in the presence of noradrenaline and sympathetic fibers in the spleen with increased hypothalamic–pituitary axis (HPA) activity. Notably, the difference is pronounced in males, who present higher parasitemia and mortality rates than females [9].

The host’s behavioral and cognitive performance is also affected by parasitic infections, and the evidence highlights the effect on memory tasks. Many parasites have been shown to worsen memory performance, including cases where the infection is not located in the central nervous system (CNS). In mice, infection with the intestinal nematode *Heligmosomoides polygyrus* causes a poorer spatial learning performance, with decreased acquisition and retention in the water maze task in a dose–response model [10]. Similarly, male rats that were heavily infected with *Toxocara canis* showed a reduced ability to solve complex maze problems [11]. Meanwhile, mice with toxocariasis also performed worse on tests relating to exploration, activity, learning, and motor coordination [12].

Although cytokines are inflammation mediators, they are essential for homeostasis and, hence, are present in healthy tissue. Particularly, in the brain, cytokines are important regulators of the synapses [13] and brain plasticity, playing a role in learning and memory. This can be seen in multiple mechanisms associated with the acquisition, retention, maintenance, and potentiation of memories. Cytokines participate in these processes at basal concentrations [14]. As reviewed in the work of Bourgognon and Cavanagh, 2020 [14], IL-1β is associated with spatial and contextual memory, while TNF-α and IL-6 are associated with spatial memory. However, if their levels are modified for critical prolonged times, for example in a chronic infection, the presence of cytokines can be detrimental, and different pathologies can develop.

In healthy conditions, the neurons of many structures of the brain express IL-6 at low levels, including the hippocampus, hypothalamus, and olfactory bulb [15]. These areas are essential for multiple functions. For instance, the hippocampus is critical for short-term memory, the hypothalamic nucleus coordinates hormone regulation, and the olfactory bulb is important for olfactory memory and mating. Therefore, all these functions can be altered upon any immune disruption.

When there is a stress stimulus, such as systemic inflammation, immune cells of the brain are recruited, and the cytokine levels increase [15]. In the brain, the microglia and astrocytes have immune functions that are activated by IL-6 and TNF-α. Indeed, the injury of a peripheral nerve triggers the elevation of TNF-α in serum, in cerebrospinal fluid (CSF), and in hippocampus tissue, even 40 days after the injury, in correlation with impaired short-term and working memory [16]. Similarly, high TNF-α levels in a model of sepsis induce memory deficits that are diminished after the administration of a TNF-α inhibitor [17].

The intraperitoneal *Taenia crassiceps* (*T. crassiceps*) infection model has been used to understand cysticercosis and the complex neuroimmune endocrine interactions under a local infection [18,19,20,21]. In this model, the parasite develops inside the peritoneal cavity of Balb/c mice, and we did not find any parasites in other organs. Interestingly, the circulating sexual hormone levels were altered dimorphically. In males, estradiol increased up to female levels, while testosterone decreased up to 15%, suggesting a feminization process as the infection progresses. Sexual behavior was also diminished in both sexes [19,22]. In female mice, the intraperitoneal chronic infection reduced the primordial and primary follicles and increased the number of atretic follicles, the ovarian P450C17, the P450-aromatase, and the serum E2 concentration [23]. This tendency shows how distant local infection can lead to systemic responses, including behavioral changes, which could benefit the parasite infection [24].

Previously, we reported that short-term memory performance is worse in infected animals. In the object recognition task, chronically infected mice spent less time exploring the new object; although both sexes were affected, the infected female showed a significant short-term memory decrease. In the same study, the cytokines and neurotransmitters of the hippocampus were quantified, showing dimorphic alterations. In females, we found an increment in IL-4, while there was a decrease in males. Additionally, the neurotransmission was altered, with a reduction in serotonin in the females and an increment in the males. In the hippocampal area, males also showed more norepinephrine levels [25]. In this work, we focus on three brain areas, the OB, hypothalamus, and hippocampus, to complement previous studies.

## 2. Materials and Methods

### 2.1. Ethics Statement

The care and experimentation were carried out according to the guidelines established in the Mexican regulation (NOM-062-ZOO-1999), which is under strict adherence to the recommendations of the Guide Care and Use of Laboratory Animals of the National Institutes of Health (NIH and The Weatherall Report). All the procedures with the animals were evaluated by the Animal Care and Use Committee (CICUAL) at the Instituto de Investigaciones Biomédicas with ethical approval permit number 2009-16.

### 2.2. Animals

Male and female Balb/cAnN (H2-d) inbred mice were obtained from Harlan (Mexico City, Mexico) and housed in the animal care facilities at UMB (Unidad de Modelos Biologicos) at the Instituto de Investigaciones Biomédicas (UNAM). The animals were cared for in the facilities at 22 °C with 12-h dark–light cycles. The mice were fed with Purina Diet 5015 (Purina, St. Louis, MO, USA) and sterile tap water ad libitum.

### 2.3. Parasitic Infection

*T. crassiceps* larvae (fast-growing ORF strain) from 3- to 6-month-infected female donor mice were used to infect male or female mice. Ten non-budding larvae with approximately 2 mm in diameter were suspended in 0.3 mL sterile phosphate-buffered saline (0.15 M NaCl, 0.01 M sodium phosphate buffer, pH 7.2). Subsequently, the larvae were intraperitoneally injected into 42-day-old male or female mice using a 0.25-gauge needle. We used five animals per group, and the experiment was duplicated.

### 2.4. Study Design

For 16 weeks, five non-infected or infected mice per sex were housed at the UMB (Unidad de Modelos Biológicos) at the Instituto de Investigaciones Biomédicas (UNAM). All mice were humanly euthanized by cervical dislocation after anesthesia with pentobarbital (Pfizer, Mexico City, Mexico). Immediately, the hippocampus, hypothalamus, and olfactory bulb were collected and frozen until determination. The neurotransmitter levels (norepinephrine, dopamine, epinephrine, serotonin) were determined by HPLC, while the cytokine expression (IL-1β, IL-4, IL-6, TNF-α, IFN-γ) was determined by PCR test.

### 2.5. Parasite Load

After the mice were euthanized, the parasites were collected thoroughly rinsing the peritoneal cavity with PBS separately for each mouse. The parasites were exclusively located in the peritoneal cavity.

### 2.6. Neurotransmitter Measurements

The hippocampus, hypothalamus, and olfactory bulb from infected or uninfected male or female mice were located and excised according to “*The mouse brain in stereotaxic coordinates*” [26] after euthanasia, immediately frozen, and collected in 1.5 mL microcentrifuge tubes. We precipitated the proteins by adding 1.5 M PCA (Perchloric acid) to the tissue. Later, the samples were sonicated until the tissue was entirely homogenized. Subsequently, the samples were centrifuged at 14,000 rpm for 15 min at 4 °C. The pellets were used to determine the protein concentration, while the supernatants were used to assess the neurotransmitter levels: NE (norepinephrine), DA (dopamine), EP (epinephrine), 5-HT, (serotonin). We used reverse-phase chromatographic analysis, with a Waters Spherisorb ODS2 C18 column, (80 Å, 5 μm, 4.6 × 250 mm) in the HPLC system composed of a Jasco PU-2085 pump, AS-2057 autosampler with an Antelec Leyden Decade II electrochemical detector, controlled by Millenium 32 software. The separation of analytes was performed at 30 °C adding the mobile phase (5% of acetonitrile in a buffer solution [12.16 mM citric acid, 11.60 mM (NH_4_)_2_HPO_4_, 2.34 mM sodium octyl sulphonate, 3.32 mM dibutyl phosphate amine, and 1.11 mM sodium EDTA] at isocratic conditions with a flow rate of 1 mL/min. The acquisition conditions for the detections were as follows: range 1 nA, filter 0.005 Hz, Eox 0.60 V, basal 0.001 V, Ic 2.72 nA. The retention times of the eluted neurotransmitters in the chromatogram were NE (5 min), EP (6 min), DA, and 5-HT (18.5 min).

### 2.7. Cytokine Expression

IL-1β, IL-4, IL-6, TNF-α, and IFN-γ were determined by PCR. The hippocampi, olfactory bulb, and hypothalamus from control or infected mice of both sexes were extracted and treated by the TRIzol reagent extraction method (Gibco-BRL, Grand Island, NY, USA) to isolate total RNA. Each tissue was dissolved and homogenized at low speed (Brinkman Polytron homogenizer) using TRIzol reagent (1 mL/0.1 g tissue) and chloroform (0.2 mL/mL TRIzol). After this procedure, the samples were centrifuged for 10 min at 14,000× *g* to recover the aqueous phase. Once obtained, we added isopropyl alcohol to precipitate the RNA and washed it with 75% ethanol. Finally, we dissolved the precipitate in RNase-free water. The RNA shrinkage and purity were determined by absorbance at 260 nm and electrophoresis in 1.0% denaturing agarose gel and 2.2 M formaldehyde. The total RNA from the tissues was reverse transcribed, and by PCR and primer design from Gene Databank (NCBI, NIH), the specific amplification of the IL-1β, IL-4, IL-6, TNF-α, and IFN-γ gene sequences was obtained. 

Once we had the total RNA, we took 1 μg from each tissue and incubated it for 60 min at 37 °C with a 50 mM mixture of each dNTP, 0.05 μL of oligo primer (dT) (Gibco-BRL), and 400 units of M-MLV reverse transcriptase (Applied Biosystems, Boston, MA, USA). cDNA (10 μL) was mixed with 5 μL of 10x PCR buffer (University Biotechnologies, Mexico City, Mexico), 1 mM MgCl, 0.2 mM of each dNTP, 0.05 μL of each primer, and 2.5 units of Taq DNA polymerase (University Biotechnologies); they were electrophoresed on a 2% agarose gel. We used a molecular weight marker 100 bp ladder (Gibco-BRL). The bands were visualized using ethidium bromide. RNA, cycling, and temperature curves for each gene were performed to determine whether all reactions were in the exponential amplification phase. Each PCR band was quantified by the densitometric scanning of several autoradiograms at various exposures and expressed as the ratio of the signal of the target gene to that of the 18S Ribosomal RNA gene. Table 1 shows the oligonucleotide sequences used for cytokine expression and the temperature cycles used to amplify after prior denaturation at 95 °C for 5 min; likewise, the genes had an extra extension to 72 °C for 10 min.

### 2.8. Statistical Analysis

We performed two independent experiments with five animals per condition. The results are presented as the mean ± standard deviation and analyzed with Prism 5 (Graph-Pad Software Inc., La Jolla, CA, USA) The statistical analysis was applied with a one-way ANOVA test followed by a Tukey post hoc test.

## 3. Results

### 3.1. The Number of Parasites in the Peritoneal Cavity Was Higher in Females than Males

**Figure 1:**
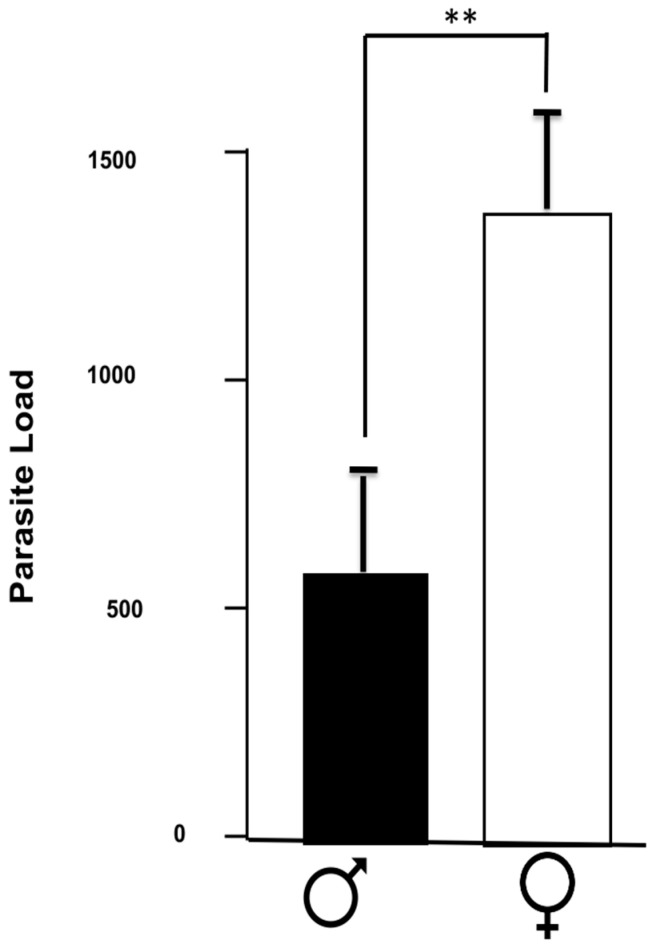
Parasite load of infected mice. The number of parasites found in male and female mice infected with *T. crassiceps* in the peritoneal cavity is indicated. The values are the mean ± SD. ** *p* < 0.01 compared to control (uninfected age-matched) mice.

As previously described, the susceptibility to *T. crassiceps* infection is dimorphic, as shown by the parasite load in the infected mice groups (Figure 1). The female mice had more than two times more parasites than the infected males. It is noteworthy that no parasites were detected outside the peritoneal cavity.

### 3.2. The Hippocampal Neurotransmitter Concentration Is Altered in a Dimorphic Way by the Intraperitoneal T. crassiceps Chronic Infection

**Figure 2:**
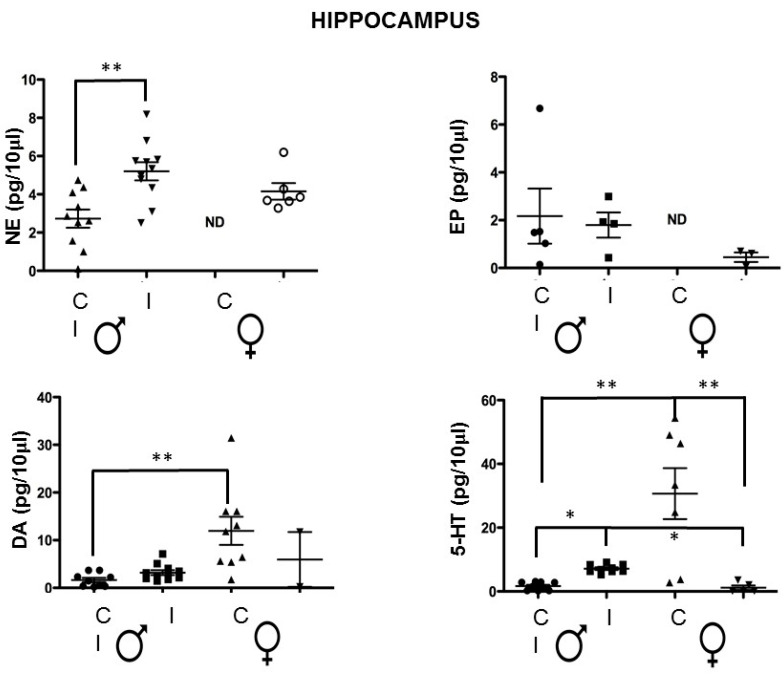
Neurotransmitter concentration in the hippocampus of the control vs. *T. crassiceps*-infected male or female mice. The values are the mean ± SD. * *p* < 0.05, ** *p* < 0.01 compared to control (uninfected age-matched) mice. NE: norepinephrine. EP: epinephrine. DA: dopamine. 5-HT: 5-hydroxytryptamine or serotonin. ND: non-detected. Control Male (Circle); Infected Male (Square); Control Female (Triangle); Infected Female (Inverted triangle).

We analyzed the expression of norepinephrine, epinephrine, dopamine, and serotonin in the hippocampus, where we found statistical differences between the control and infected mice in a dimorphic manner. Serotonin levels decreased in the infected females and increased in males. Norepinephrine levels increased in both sexes but were significantly different only in males (Figure 2). Additionally, dopamine showed a dimorphic concentration in the control groups, with higher levels in females than males. In contrast, dopamine levels were non-detected in most of the infected male group sample, but the infection did not modulate this neurotransmitter.

### 3.3. The OB Neurotransmitters Concentration Is Altered in a Dimorphic Way by Intraperitoneal T. crassiceps Chronic Infection

The olfactory bulb participates in working memory and mating depending on olfaction [21]. The olfactory bulb processes odor signals, including pheromones that mediate attraction to the males and volatile urine compounds that allow the identification of infected and uninfected male mice [22], guiding the mate choice [23]. By its connection with the amygdala [24], the nucleus accumbens [25], and the hypothalamus, there is a hub of social information processing that determines behavior changes. In addition, the olfactory bulbs’ oscillatory activity modulates the information process to other regions during spatial working memory [26,27]. Since infection causes changes in reproductive behaviour, we were interested in determining the neurotransmitters in this area [19,22]. In OB, females’ norepinephrine was not detected, regardless of their infection status, and we did not find differences in males.

**Figure 3:**
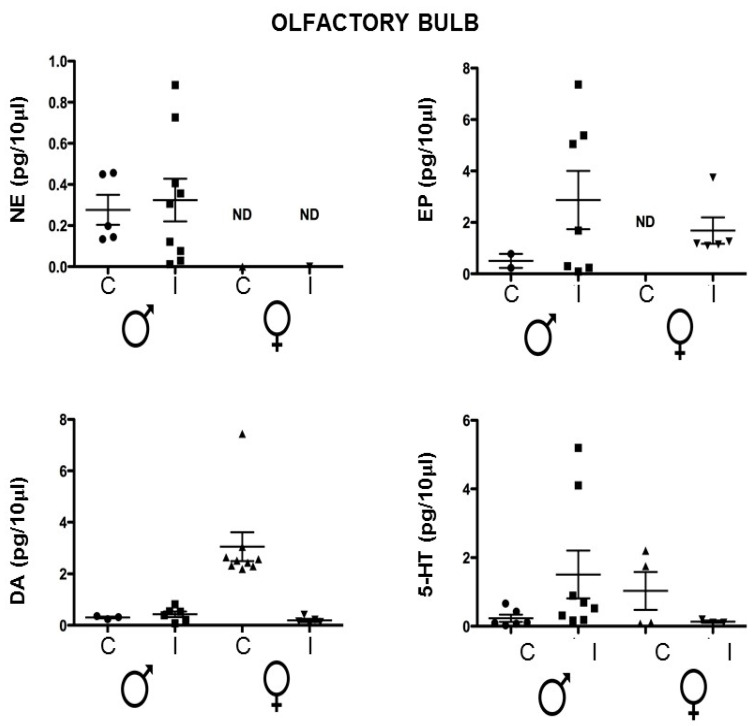
Neurotransmitter quantification in the olfactory bulb of the control vs. *T. crassiceps*-infected male or female mice. ND: non-detected. The values are the mean ± SD. Compared to the control (uninfected age-matched) mice. NE: norepinephrine. EP: epinephrine. DA: dopamine. 5-HT: 5-hydroxytryptamine or serotonin. ND: non-detected. Control Male (Circle); Infected Male (Square); Control Female (Triangle); Infected Female (Inverted triangle).

**Figure 4:**
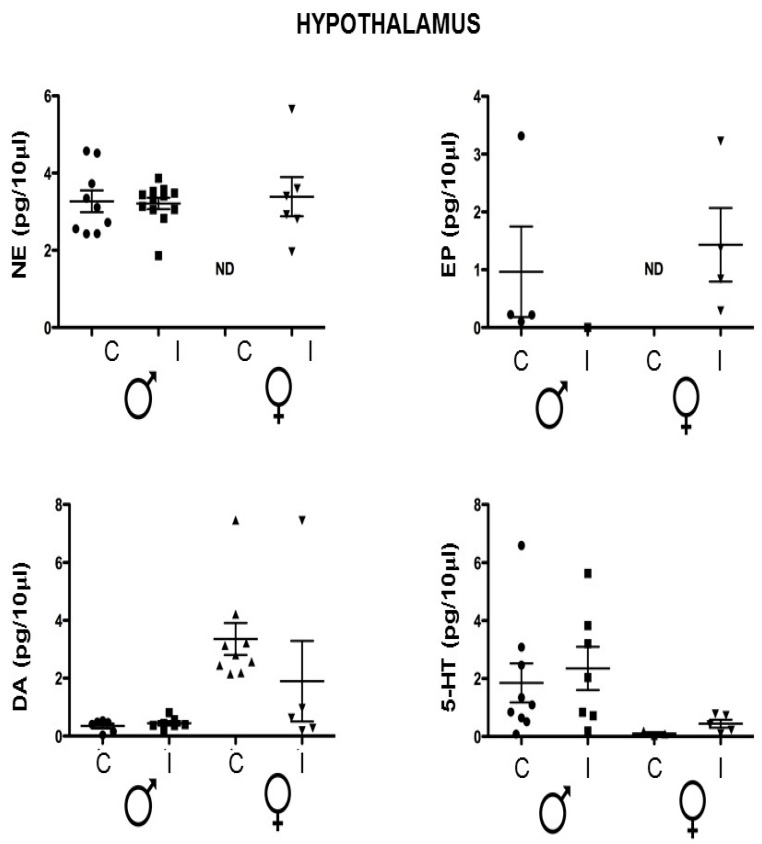
Neurotransmitter levels in the hypothalamus of the control vs. *T. crassiceps*-infected male or female mice. The values are presented as the mean ± SD. NE: norepinephrine. EP: epinephrine. DA: dopamine. 5-HT: 5-hydroxytryptamine or serotonin. ND: non-detected. Control Male (Circle); Infected Male (Square); Control Female (Triangle); Infected Female (Inverted triangle).

Epinephrine was not detected in the female control group, while it was elevated in the infected group. In males, the levels seem to be augmented, although there was no statistical significance. In the case of dopamine, the concentration was lower in the infected females, but not significantly. The serotonin levels were similar between groups (Figure 3). In the hypothalamus, the norepinephrine and the epinephrine male groups showed no differences. In the females, the control levels were non-detected, while the levels in the infected animals were detectable, indicating an increase like the groups in the hippocampus. There were also no differences in the dopamine and serotonin levels (Figure 4).

### 3.4. Hypothalamus Neurotransmitters Are Not Affected by the T. crassiceps Infection

The hypothalamus contains gonadotropin-releasing hormone (GnRH) neurons, which receive multiple signals mediated by neurotransmitters such as serotonin and norepinephrine, among others, modulating the release of the GnRH to the pituitary and regulating the LH/FSH release. This finally modulates the sexual hormone production by the gonads [19]. Additionally, the hypothalamus is involved in male behavior, which tends to be modified as the animal tries to avoid the infected pairs [20]. Since we found that males increase estradiol production and decrease testosterone, it is likely that the hypothalamus will be studied to investigate whether neurotransmitters are also altered in a dimorphic manner.

None of the quantified neurotransmitters showed any difference between the control and the infected mice (Figure 4). Interestingly, there are dimorphic levels among the control groups that are not evident among the infected ones, although there is no statistical significance. The NE and EP in female were not detectable; meanwhile, some of the male hypothalami showed detectable levels of the neurotransmitter. The dopamine in the females without infection was higher, and the 5-HT was lower than in the males.

### 3.5. The Inflammatory Profile Is Different in Each Brain Area, and It Depends on the Sex

The cytokines IL-1β, IL-4, IL-6, TNF-α, and IFN-γ were measured in the hippocampus, olfactory bulb, and hypothalamus, since it has been observed that the brain senses intraperitoneal infections and possesses the capacity to respond to them. Our results confirmed neuroinflammation through cytokines dimorphically in the hippocampus and the olfactory bulb, as previously reported by the group with the same model evaluating the cytokines at 4, 8, and 16 weeks of infection [27]. The hippocampus is essential in retaining object recognition memory, which is implied in short-term memory, as is the perirhinal cortex [28]. Not only neurotransmitters but also cytokines such as IL-1β, IL-6, and TNF-α are implicated in learning and memory [29].

**Figure 5:**
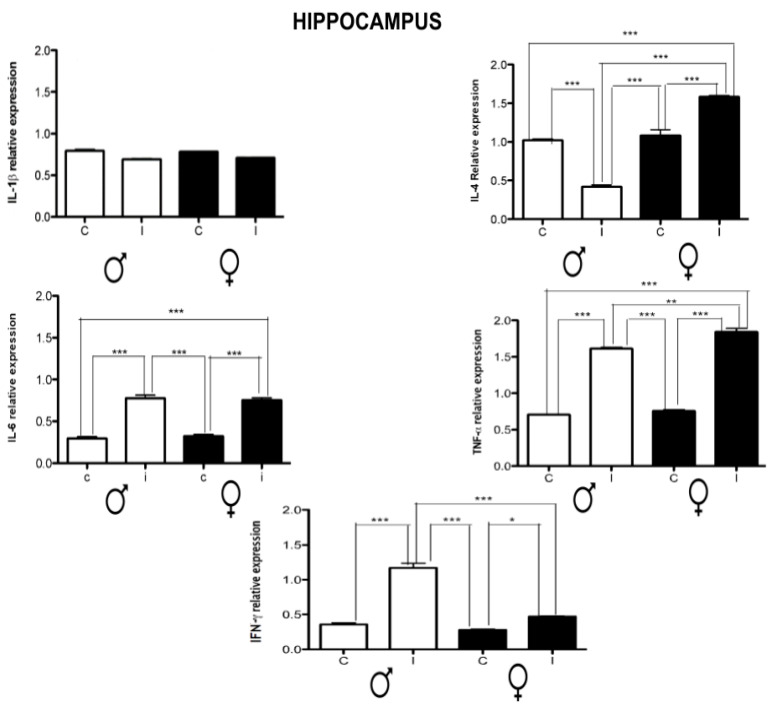
Effect of chronic *T. crassiceps* infection in the expression of IL-1β, IL-4, IL-6, TNF-α, and IFN-γ in the hippocampus of the control vs. infected male and female mice. The values are the mean ± SD. * *p* < 0.05, ** *p* < 0.01, *** *p* < 0.001 compared to control (uninfected age-matched) mice.

The hippocampal levels of cytokines were dimorphic in IL-4 and IFN-γ. For females, IL-4 was up-regulated while for males, it was down-regulated. IFN-γ was up-regulated in both sexes. However, for males, it was more than two times higher than for females. Both infected groups had IL-6 and TNF-α up-regulation levels in this area. For IL-1β, there were no significant changes between the controls and infected groups (Figure 5).

**Figure 6:**
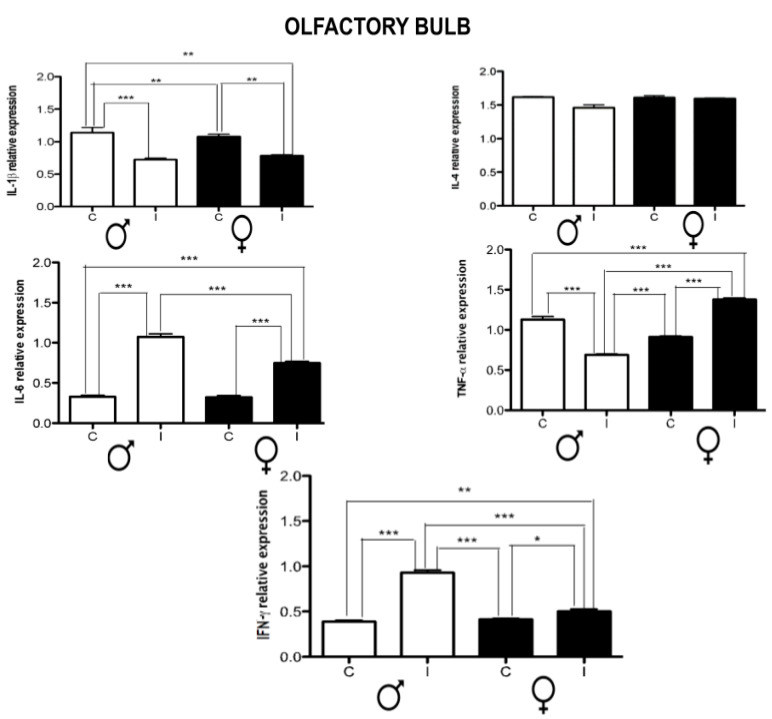
Effect of chronic *T. crassiceps* infection in the expression of IL-1β, IL-4, IL-6, TNF-α, and IFN-γ in the olfactory bulb of the control vs. infected male and female mice. The values are the mean ± SD. * *p* < 0.05, ** *p* < 0.01, *** *p* < 0.001 compared to control (uninfected age-matched) mice.

On the other hand, olfactory bulb neuroinflammation presented a dimorphic regulation only in TNF-α, which was up-regulated in females, while in males, it was down-regulated. In both cases, there was an up-regulation of IL-6 and IFN-γ. Regarding IL-1β there was down-regulation in both sexes, and there were no significant changes in all cases (Figure 6).

**Figure 7:**
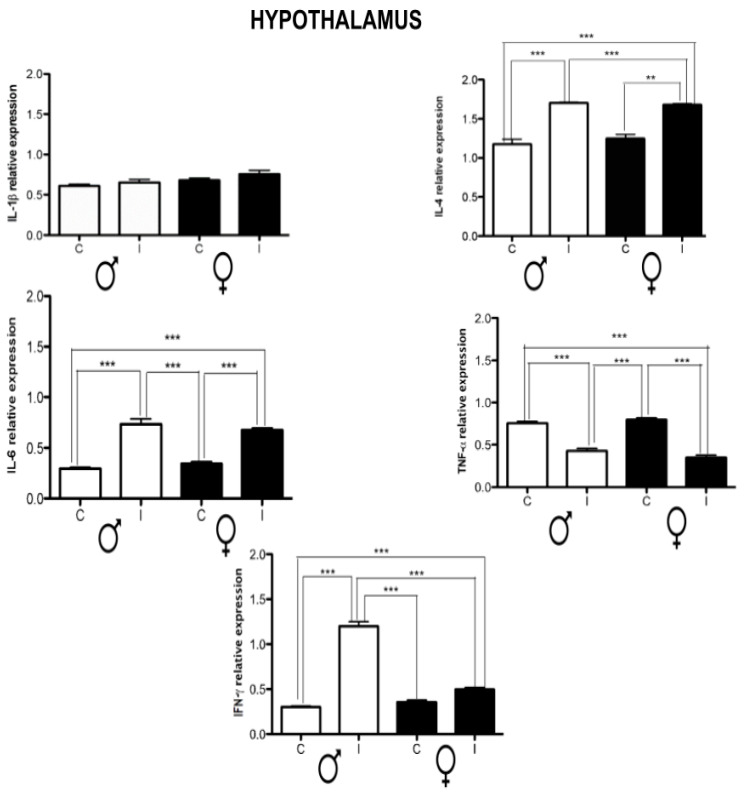
Effect of chronic *T. crassiceps* infection in the expression of IL-1β, IL-4, IL-6, TNF-α, and IFN-γ in the hypothalamus of the control vs. infected male and female mice. The values are the mean ± SD. ** *p* < 0.01, *** *p* < 0.001 with respect to control (uninfected age-matched) mice.

Finally, in the hypothalamus, there was no dimorphic neuroinflammation. All cytokines were up-regulated, except for IL-1β and TNF-α, which showed the opposite effect. Of note, IFN-γ was up-regulated almost threefold in infected females (Figure 7).

## 4. Discussion

The description of the communication among the different molecules and cells in the neuroimmunendocrine network during parasitic infections may contribute to a better understanding of the course of the disease. We have studied the role of hormones, cytokines, and neurotransmitters during acute and chronic infection with the parasite *T. crassiceps* and found that changes in the immune response activated locally (n the peritoneal cavity of infected mice) may induce changes in the brain, causing alterations in mood and behaviour, as observed in other animal models and children. In this model, the females are more susceptible to parasitosis, while the males are more resistant at the onset of the infection. However, the chronic infection provokes a feminization process in which the systemic estradiol concentration increases, and the testosterone decreases, while the parasite load augments [22]. At the same time, we registered several behavioral changes. In males, there is a loss in mating interest, loss of ejaculation response, fewer mounts, and intromission. ¡Females also show less receptivity and significant disruptions in the estrous cycle [19,22]. 

We have previously reported that short-term memory performance was decreased in infected animals with novel-object recognition, with females having worse performance. We have demonstrated that the serotonin 5-HT levels were dimorphically expressed, with females having higher levels than males. However, upon infection, the infected female mice showed a decrease in the levels of 5-HT in the hippocampus while in males, it was a slight increase [25], and these observations were consistent in this work. Regarding norepinephrine, basal levels in females were not detected, but they were augmented in infected animals; meanwhile, the male controls presented lower levels that increased significantly in the infected males. Additionally, we did not observe differences in the neurotransmitters, 5-HT, dopamine, norepinephrine, and epinephrine levels in olfactory bulbs and the hypothalamus, suggesting that these areas are not crucial to mood and behavior, but as mentioned, they may have a role in mating and sexual behavior.

Since neuroimmune regulation has been widely described, we looked for differences in the expression of pro-inflammatory cytokines in the hippocampus, demonstrating that the brain’s immune system responds to peritoneal infection through neuroinflammation [25]. The cytokines in other cerebral areas, including the frontal and lateral cortex, olfactory bulbs, hypothalamus, and preoptic area, are altered in an area-specific manner during intraperitoneal infection, and in some cases, the differences are dimorphic [27]. As cytokines are immune systemic mediators capable of modulating neurotransmission [28], we hypothesize that the concentration of neurotransmitters will be altered according to the levels of cytokines in each area. As explained above, the hippocampus is essential to short-term memory, and the hypothalamic nucleus coordinates hormone regulation; meanwhile, the olfactory bulb is important to olfactory memory and mating. These are behaviors affected by the infection [29,30,31].

**Figure 8:**
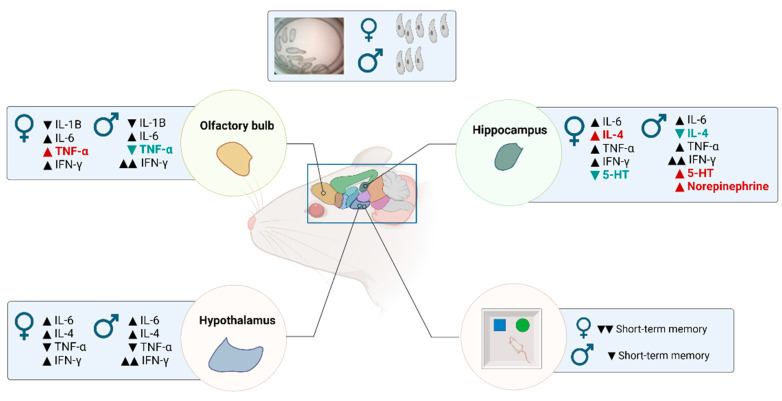
Cytokine and neurotransmitter level changes after a chronic infection with *T. crassiceps* in the olfactory bulb, hippocampus, and hypothalamus. The parasite load was higher in males; meanwhile, the short-term memory deficit was worse in females. ▲: Increment infected vs. control. ▼: Decrement infected vs. control. Red and blue colors mean a dimorphic change. This figure was designed by using software purchased from Biorender. Permission for use is given via Biorender [38].

Here, we showed that, in chronic infection, the males presented a lower parasite load than infected females, as expected. The neuroinflammatory profile of the different brain areas showed a different neuroinflammatory profile. In the olfactory bulbs, IL-6 and IFN-γ were elevated in both sexes, while IL-1β was reduced. The TNF-α levels were dimorphic, with elevated levels in females and decreased levels in males. In the hypothalamus, IL-6 and IFN-γ were also elevated; meanwhile, TNF-α and IL-4 were diminished in both sexes. Finally, in the hippocampus, IL-6, and TNF-α were elevated in both sexes. In the male-infected mice, the IFN-γ was elevated almost three times compared to a small but significant increase in females. These observations support our previous reports [25,27]. On the other hand, we found that the IL-4 hippocampal levels were lower in the male controls than in females, in which IL-4 was elevated (Figure 8).

Systemic cytokine inhibition has been associated with changes in neuroinflammation. This is the case of TNF-α inhibition that abolishes sepsis-induced cognitive impairment in mice by modulating different soluble mediators and neuroinflammation [17]. The increased levels of TNF-α in the hippocampus may correlate with their deficit for adequate performance, while other areas such as OB and hypothalamus may not play a determinant role in this behavior. On the other hand, locally expressed IFN-γ regulates neuronal connectivity and social behavior in the brain [32]. Since an increased expression of IFN-γ is observed in infected males in all areas analyzed, it may protect the alterations observed in cognitive performance. In contrast, we found that females who showed poorer short-term memory had a lower increment in the expression of IFN-γ associated with the infection in all brain areas and kept lower levels of this cytokine when compared to males.

Considering the literature, it has been seen that *Schistosoma mansoni* infection increases IL-4 levels at a systemic level, although there are no reports of dimorphic studies [33]. Interestingly, IL-4 plays an important role in cognition, since its impairment, caused by a lack of T cells that produce it or by the abolishment of IL-4R, is associated with cognitive impairment in learning tasks on the Morris water maze (MWM) [34]. This observation does not support our findings that females showed behavioral deficits but had higher levels of IL-4 in both the hypothalamus and hippocampus during chronic infection. However, other cytokines such as TNF-α and IFN-γ may have a preponderant role in this model.

For a long time, we have used the intraperitoneal *T. crassiceps* infection as an animal model to study the neuroimmune endocrine network. In this model, parasites do not invade any other areas than the peritoneal cavity, but they are capable of causing a systemic response associated with considerable hormonal and behavioral changes in a dimorphic manner [35]. The observations in this work support that changes induced by the infection have an impact on the neuroimmune profiles depending on the brain area that was analyzed, with the hippocampus most associated with cognition. However, this does not rule out that there may be changes in the function of specific neuronal tissues or any other neurotransmitters in the OB or the hypothalamus.

Finally, changes observed locally in the brain may be caused by different pathways: through the HPA axis and the vagal innervation or by the systemic circulation of cytokines crossing the BBB and reaching different brain areas. Vagal innervation has been associated with changes in the immunoendocrine profile in other infections, such as our model of *Trichinella spiralis* in which, by eliminating this communication through vagotomy, infected animals decreased the neuroimmune response in the brain (Personal communication). On the other hand, a systemic inflammation characterized by an increment in the levels of IL-4, IL-5, and IL-10 of infected mice [36] may reach the brain and penetrate the blood–brain barrier, which was described in the work of Van Belle et al. [37], including a permeabilization of the BBB in *T. crassiceps* infection at 10–12 weeks. Therefore, future studies on the effects of neurotransmitter modulation deserve attention.

## 5. Conclusions

In this work, we showed how a peripheral infection can provoke neuroinflammation with specific cytokine profiles in particular brain areas, which, along with the hormonal and behavioral changes reported in other studies, are an example of the synergic interaction between the immune, the endocrine, and the nervous system. Even when the infection is in the intraperitoneal cavity, there are molecular changes inside the central nervous system, mainly by the elevation of inflammatory cytokines and modifications on the hippocampal neurotransmitters 5-HT in both sexes and the NE in males, showing that the hippocampus is more vulnerable to the peripheric infection, and it correlates with the short-term memory deficit previously shown. Furthermore, it demonstrates a communication between the immune and nervous systems expanding the knowledge that we have accumulated regarding the intraperitoneal *T. crassiceps* chronic infection model and how it illustrates the complexity of the neuroimmune endocrine system function in the complex host–parasite interaction.

## Figures and Tables

**Table 1:** The oligonucleotide sequences used for cytokine expression and the temperature cycles used to amplify after prior denaturation at 95 °C for 5 min; likewise, the genes had an extra extension to 72 °C for 10 min.

Name (Size)	Forward Sequence	Reverse Sequence	Temperature Cycling			Total Cycling
IL-1β (503)	5′-tcatgggatgatgatgataacctgct	5′-cccatactttaggaagacacggatt	95 °C 30 s	57 °C 45 s	72 °C 45 s	35
IL-4 (181)	5′-cgaagaacaccacagagagtgagct	5′-gactcattcatggtgcgacttatcg	90 °C 30 s	60 °C 45 s	72 °C 50 s	30
IL-6 (638)	5′-acctggtagaagtgatgccccaggca	5′-ctatgcagttgatgaagatgtcaaa	95 °C 45 s	60 °C 55 s	72 °C 50 s	30
TNF-α (300)	5′-ggcaggtctactttggagtcattgc	5′-acattcgaggctccagtgaattcgg	90 °C 50 s	57 °C 45 s	72 °C 30 s	40
IFN-γ (247)	5′-agcggctgactgaactcagattgtag	5′-gtcacagttttcagctgtataggg	95 °C 30 s	58 °C 35 s	72 °C 40 s	35

## Data Availability

The original contributions presented in the study are included in the article. Further inquiries can be directed to the corresponding author/s.
